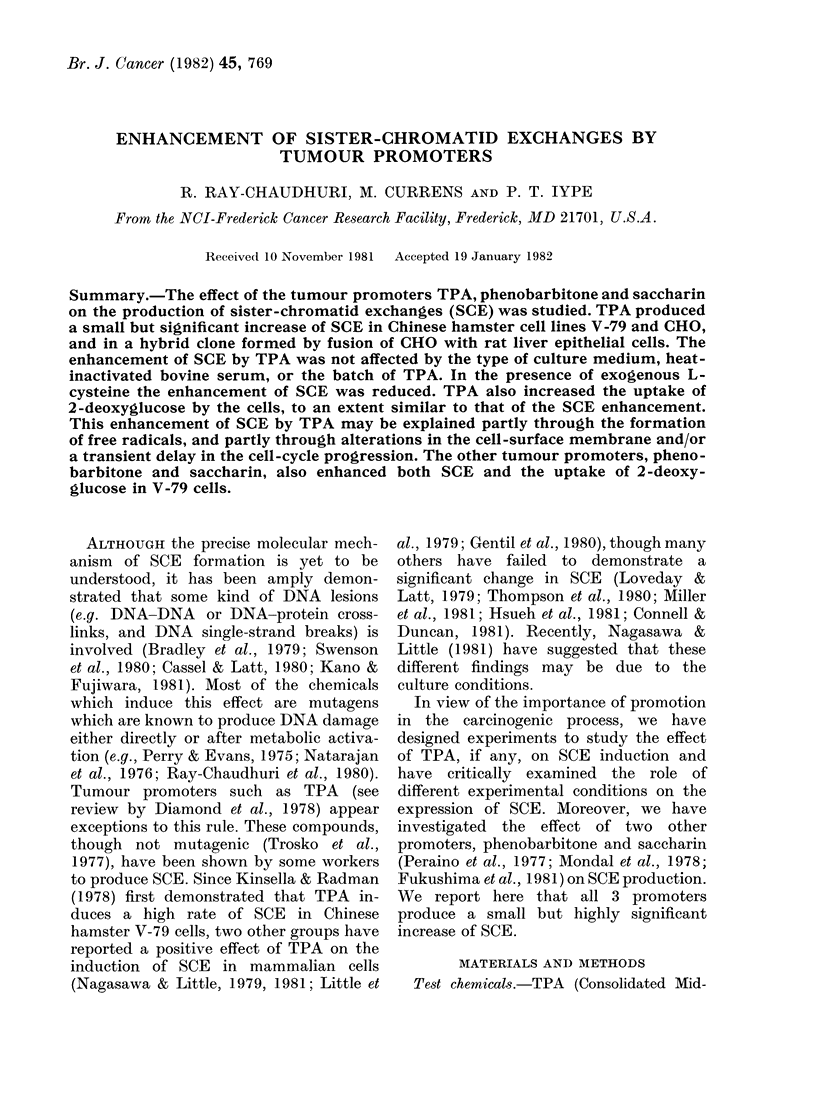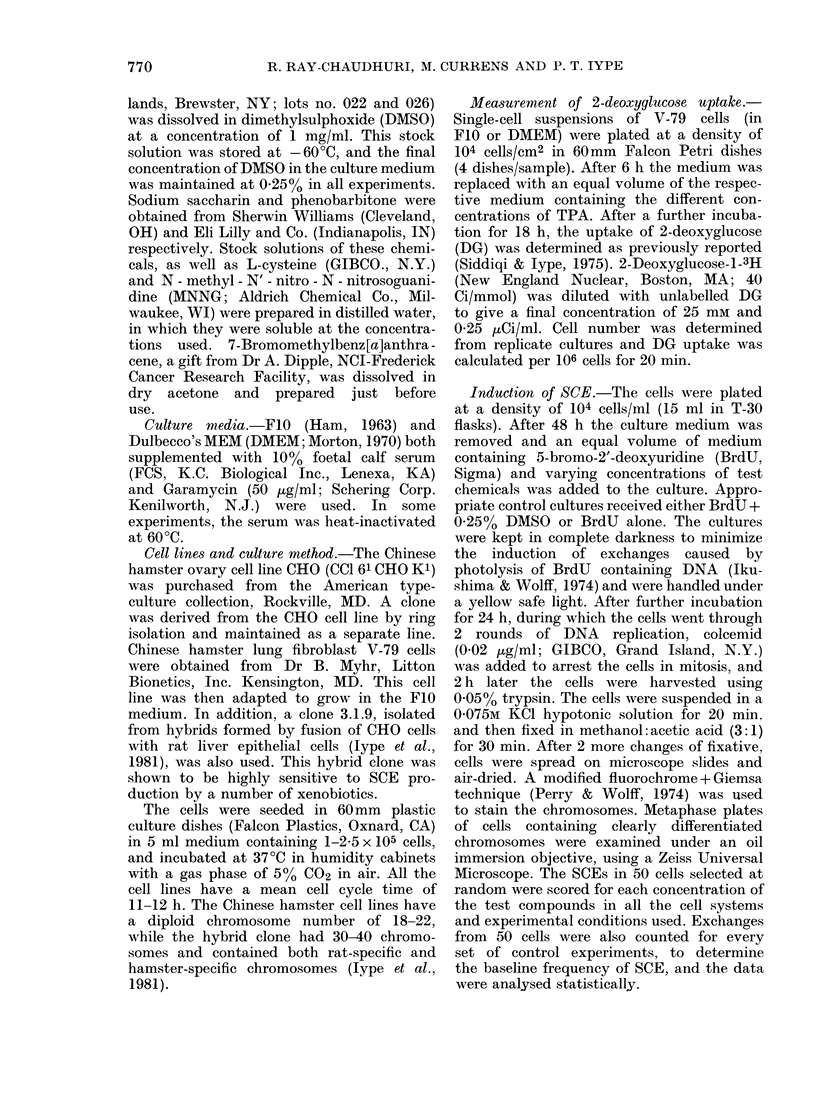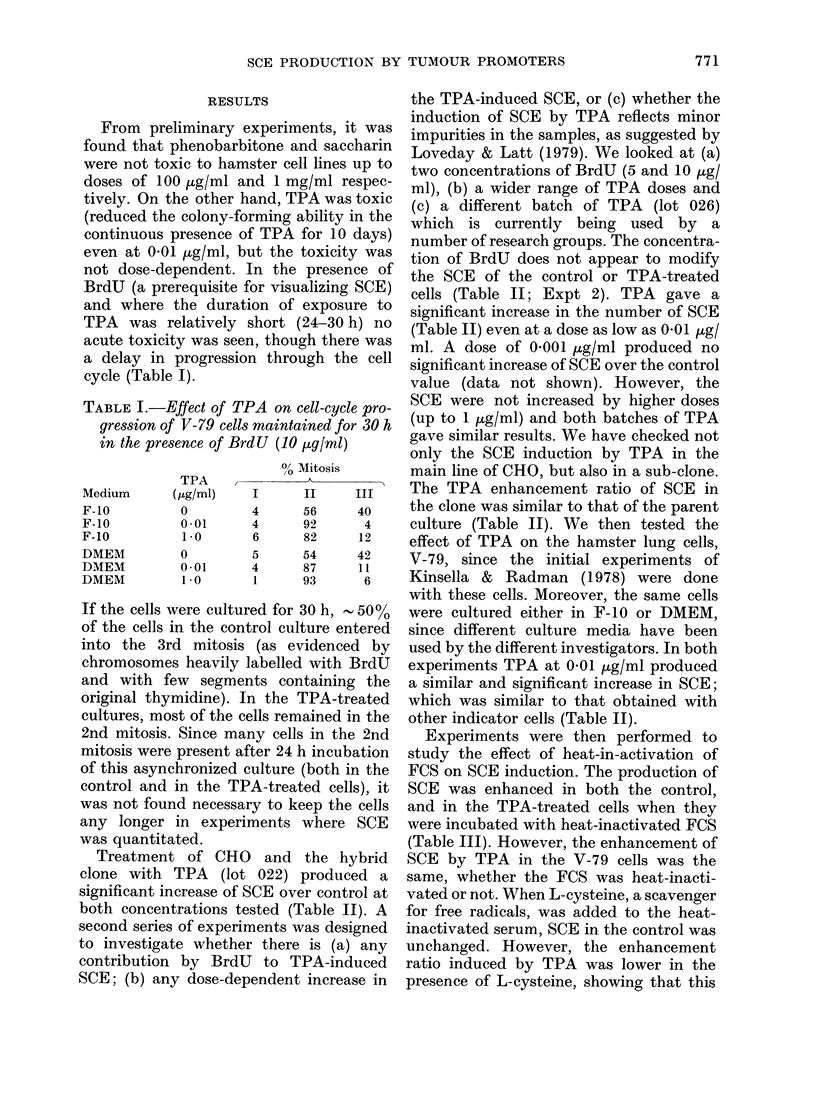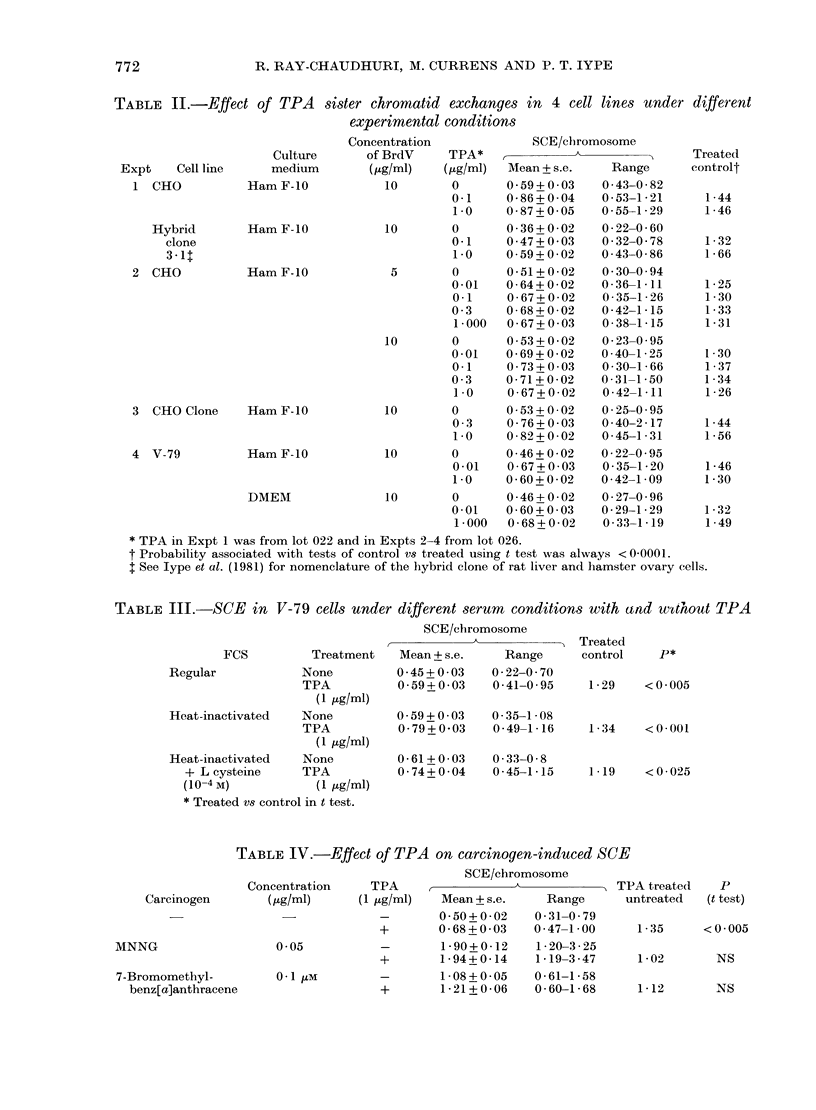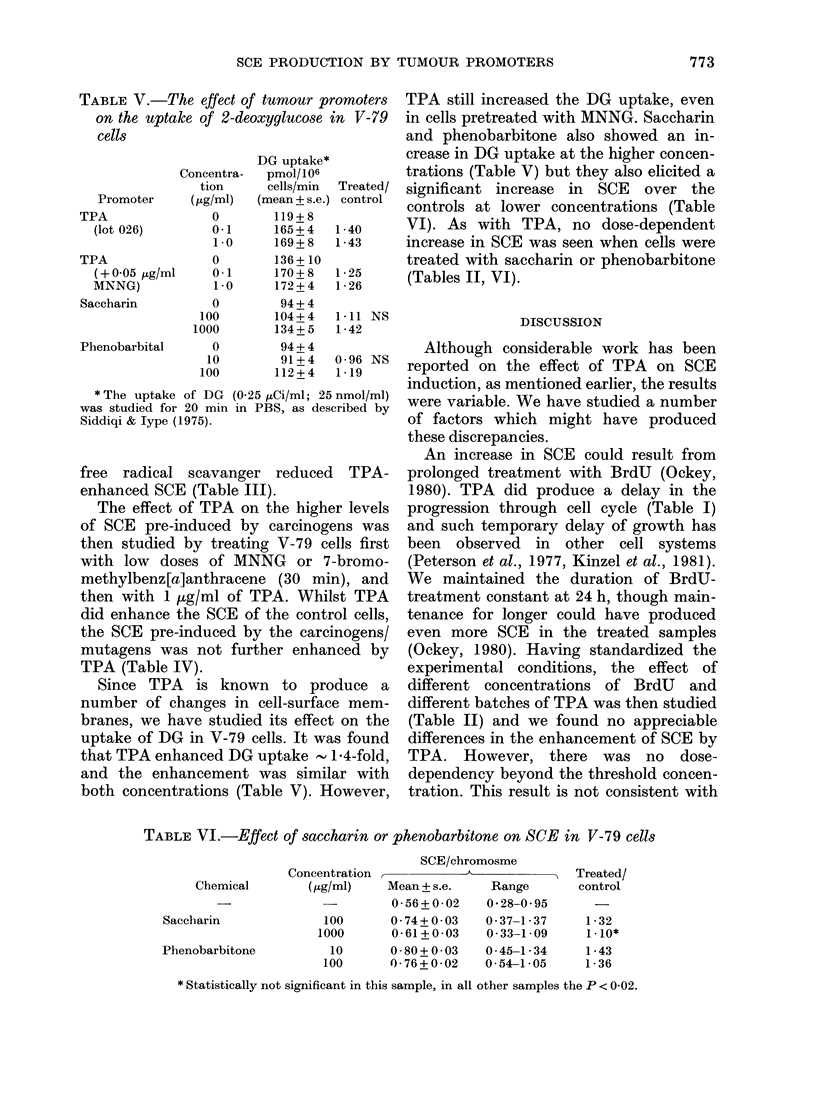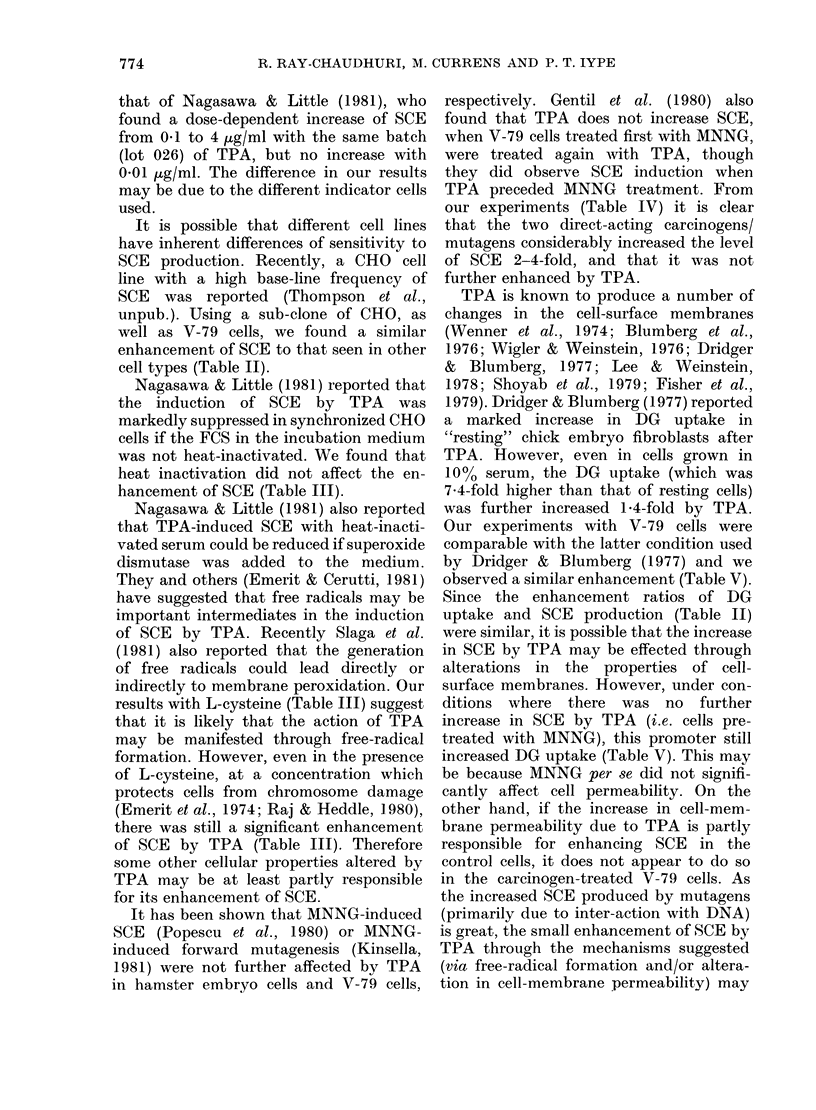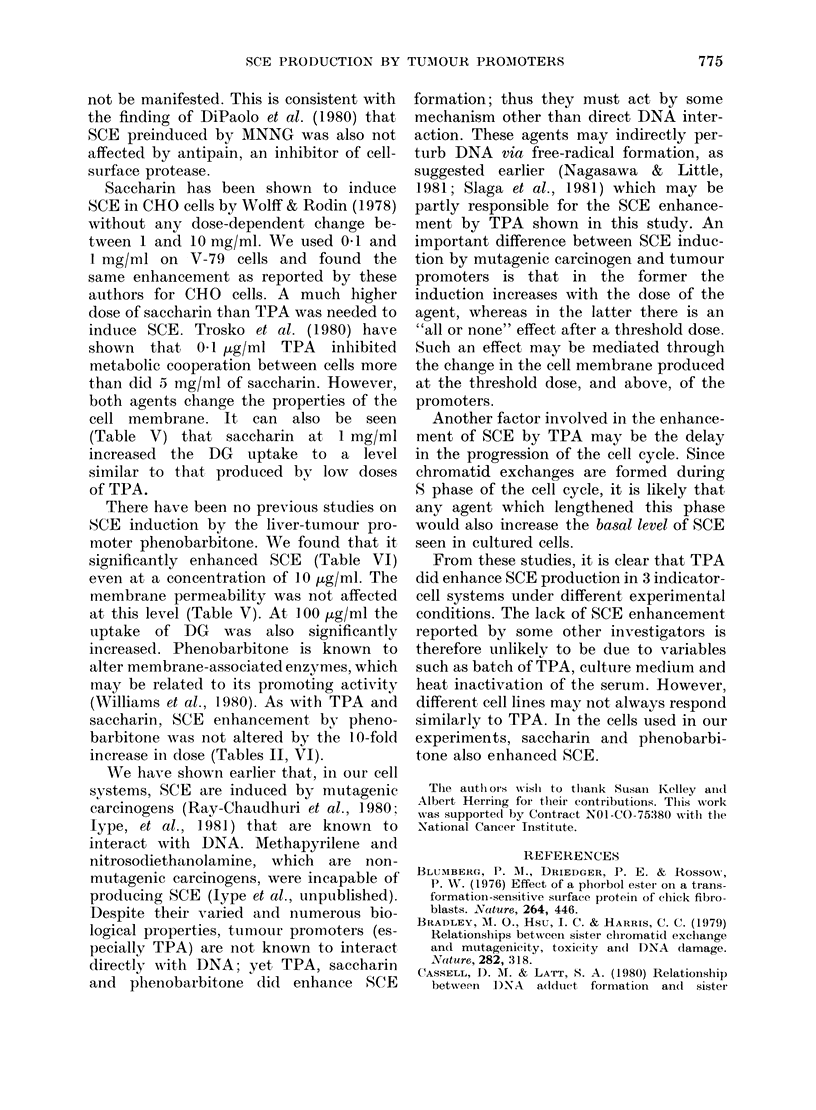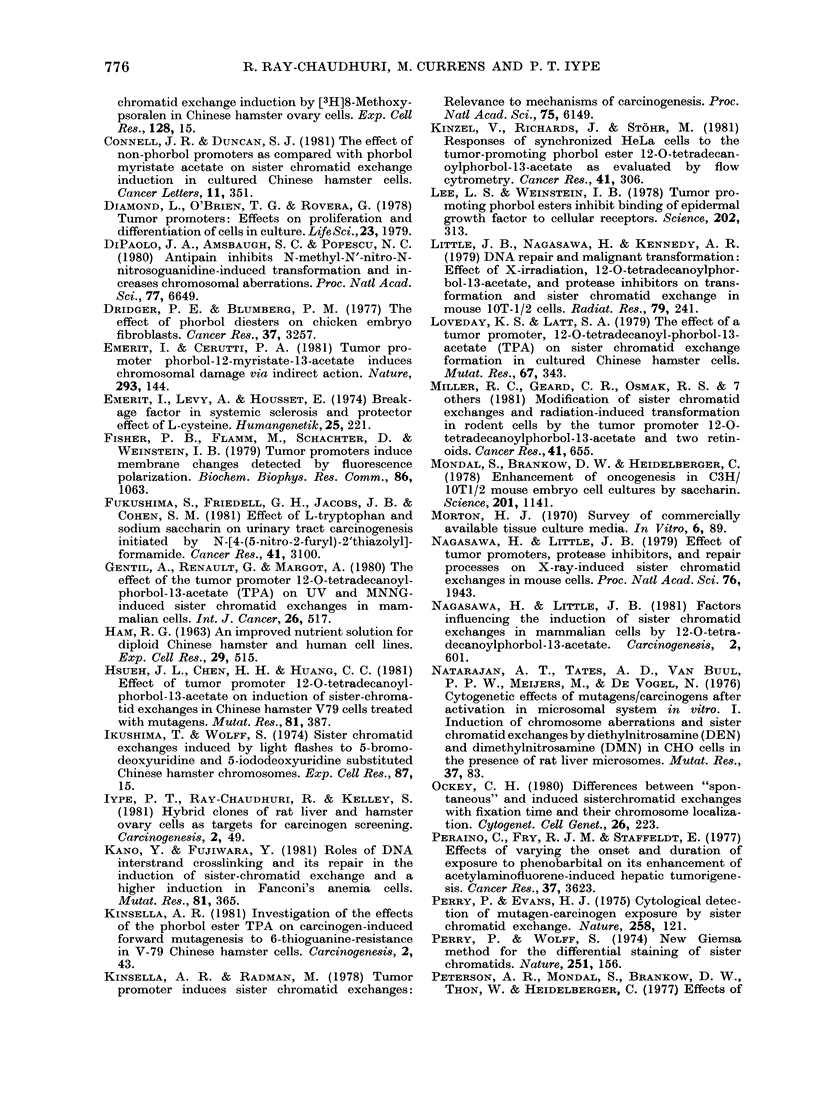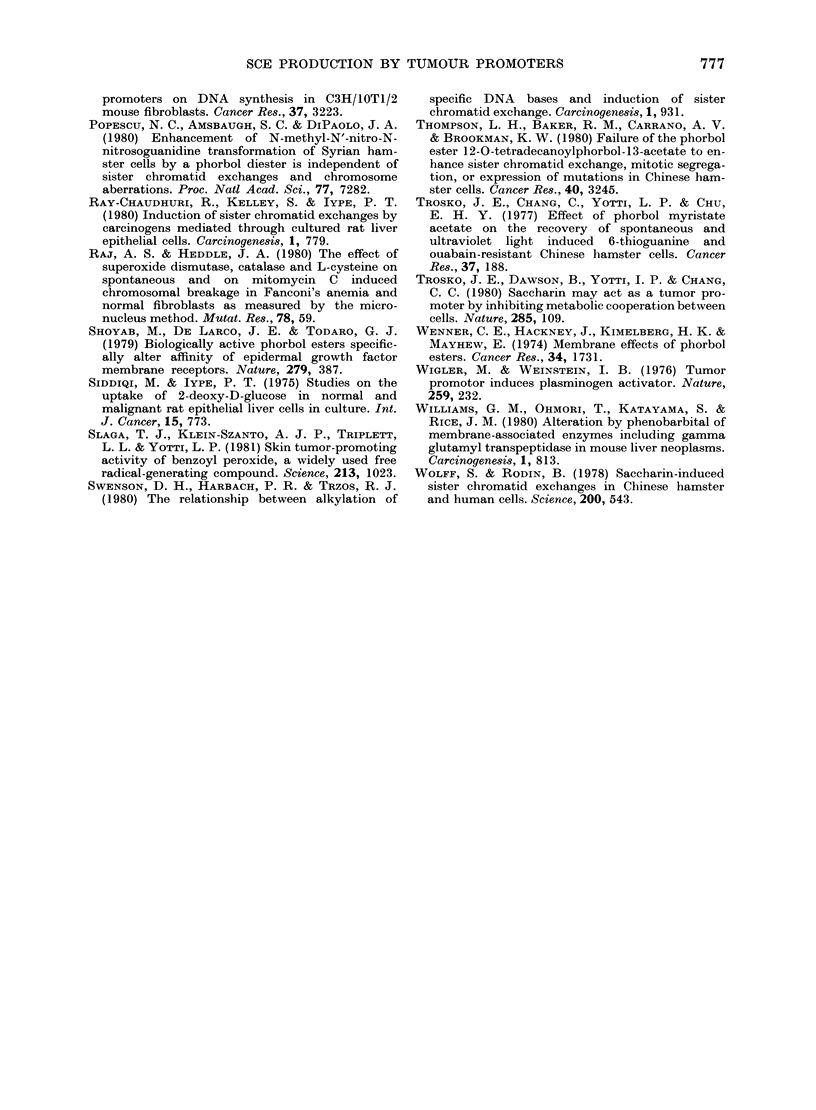# Enhancement of sister-chromatid exchanges by tumour promoters.

**DOI:** 10.1038/bjc.1982.119

**Published:** 1982-05

**Authors:** R. Ray-Chaudhuri, M. Currens, P. T. Iype

## Abstract

The effect of the tumour promoters TPA, phenobarbitone and saccharin on the production of sister-chromatid exchanges (SCE) was studied. TPA produced a small but significant increase of SCE in Chinese hamster cell lines V-79 and CHO, and in a hybrid clone formed by fusion of CHO with rat liver epithelial cells. The enhancement of SCE by TPA was not affected by the type of culture medium, heat-inactivated bovine serum, or the batch of TPA. In the presence of exogenous L-cysteine the enhancement of SCE was reduced. TPA also increase the uptake of 2-deoxyglucose by the cells, to an extent similar to that of the SCE enhancement. This enhancement of SCE by TPA may be explained partly through the formation of free radicals, and partly through alterations in the cell-surface membrane and/or a transient delay in the cell-cycle progression. The other tumour promoters, phenobarbitone and saccharin, also enhanced both SCE and the uptake of 2-deoxyglucose in V-79 cells.


*Br. J. Cancer* (1982) **45**, 769

### ENHANCEMENT OF SISTER-CHROMATID EXCHANGES BY TUMOUR PROMOTERS

R. RAY-CHAUDHURI, M. CURRENS AND P. T. IYPE

*From the NCI-Frederick Cancer Research Facility, Frederick, MD* 21701, *U.S.A.*

Received 10 November 1981 Accepted 19 January 1982

**Summary.—The effect of the tumour promoters TPA, phenobarbitone and saccharin on the production of sister-chromatid exchanges (SCE) was studied. TPA produced a small but significant increase of SCE in Chinese hamster cell lines V-79 and CHO, and in a hybrid clone formed by fusion of CHO with rat liver epithelial cells. The enhancement of SCE by TPA was not affected by the type of culture medium, heat-inactivated bovine serum, or the batch of TPA. In the presence of exogenous L-cysteine the enhancement of SCE was reduced. TPA also increased the uptake of 2-deoxyglucose by the cells, to an extent similar to that of the SCE enhancement. This enhancement of SCE by TPA may be explained partly through the formation of free radicals, and partly through alterations in the cell-surface membrane and/or a transient delay in the cell-cycle progression. The other tumour promoters, phenobarbitone and saccharin, also enhanced both SCE and the uptake of 2-deoxyglucose in V-79 cells.**

ALTHOUGH the precise molecular mechanism of SCE formation is yet to be understood, it has been amply demonstrated that some kind of DNA lesions (*e.g.* DNA–DNA or DNA–protein cross-links, and DNA single-strand breaks) is involved (Bradley *et al.*, 1979; Swenson *et al.*, 1980; Cassel & Latt, 1980; Kano & Fujiwara, 1981). Most of the chemicals which induce this effect are mutagens which are known to produce DNA damage either directly or after metabolic activation (*e.g.*, Perry & Evans, 1975; Natarajan *et al.*, 1976; Ray-Chaudhuri *et al.*, 1980). Tumour promoters such as TPA (see review by Diamond *et al.*, 1978) appear exceptions to this rule. These compounds, though not mutagenic (Trosko *et al.*, 1977), have been shown by some workers to produce SCE. Since Kinsella & Radman (1978) first demonstrated that TPA induces a high rate of SCE in Chinese hamster V-79 cells, two other groups have reported a positive effect of TPA on the induction of SCE in mammalian cells (Nagasawa & Little, 1979, 1981; Little *et al.*, 1979; Gentil *et al.*, 1980), though many others have failed to demonstrate a significant change in SCE (Loveday & Latt, 1979; Thompson *et al.*, 1980; Miller *et al.*, 1981; Hsueh *et al.*, 1981; Connell & Duncan, 1981). Recently, Nagasawa & Little (1981) have suggested that these different findings may be due to the culture conditions.

In view of the importance of promotion in the carcinogenic process, we have designed experiments to study the effect of TPA, if any, on SCE induction and have critically examined the role of different experimental conditions on the expression of SCE. Moreover, we have investigated the effect of two other promoters, phenobarbitone and saccharin (Peraino *et al.*, 1977; Mondal *et al.*, 1978; Fukushima *et al.*, 1981) on SCE production. We report here that all 3 promoters produce a small but highly significant increase of SCE.

### MATERIALS AND METHODS

*Test chemicals.*—TPA (Consolidated Midlands, Brewster, NY; lots no. 022 and 026) was dissolved in dimethylsulphoxide (DMSO) at a concentration of 1 mg/ml. This stock solution was stored at −60°C, and the final concentration of DMSO in the culture medium was maintained at 0·25% in all experiments. Sodium saccharin and phenobarbitone were obtained from Sherwin Williams (Cleveland, OH) and Eli Lilly and Co. (Indianapolis, IN) respectively. Stock solutions of these chemicals, as well as L-cysteine (GIBCO., N.Y.) and N - methyl - N′ - nitro - N - nitrosoguanidine (MNNG; Aldrich Chemical Co., Milwaukee, WI) were prepared in distilled water, in which they were soluble at the concentrations used. 7-Bromomethylbenz[*a*]anthracene, a gift from Dr A. Dipple, NCI-Frederick Cancer Research Facility, was dissolved in dry acetone and prepared just before use.

*Culture media.*—F10 (Ham, 1963) and Dulbecco's MEM (DMEM; Morton, 1970) both supplemented with 10% foetal calf serum (FCS, K.C. Biological Inc., Lenexa, KA) and Garamycin (50 μg/ml; Schering Corp. Kenilworth, N.J.) were used. In some experiments, the serum was heat-inactivated at 60°C.

*Cell lines and culture method.*—The Chinese hamster ovary cell line CHO (CCl $6^1$ CHO $K^1$) was purchased from the American type-culture collection, Rockville, MD. A clone was derived from the CHO cell line by ring isolation and maintained as a separate line. Chinese hamster lung fibroblast V-79 cells were obtained from Dr B. Myhr, Litton Bionetics, Inc. Kensington, MD. This cell line was then adapted to grow in the F10 medium. In addition, a clone 3.1.9, isolated from hybrids formed by fusion of CHO cells with rat liver epithelial cells (Iype *et al.*, 1981), was also used. This hybrid clone was shown to be highly sensitive to SCE production by a number of xenobiotics.

The cells were seeded in 60mm plastic culture dishes (Falcon Plastics, Oxnard, CA) in 5 ml medium containing $1–2·5 \times 10^5$ cells, and incubated at 37°C in humidity cabinets with a gas phase of 5% $CO_2$ in air. All the cell lines have a mean cell cycle time of 11–12 h. The Chinese hamster cell lines have a diploid chromosome number of 18–22, while the hybrid clone had 30–40 chromosomes and contained both rat-specific and hamster-specific chromosomes (Iype *et al.*, 1981).

*Measurement of 2-deoxyglucose uptake.*—Single-cell suspensions of V-79 cells (in F10 or DMEM) were plated at a density of $10^4$ cells/$cm^2$ in 60mm Falcon Petri dishes (4 dishes/sample). After 6 h the medium was replaced with an equal volume of the respective medium containing the different concentrations of TPA. After a further incubation for 18 h, the uptake of 2-deoxyglucose (DG) was determined as previously reported (Siddiqi & Iype, 1975). 2-Deoxyglucose-1-$^3H$ (New England Nuclear, Boston, MA; 40 Ci/mmol) was diluted with unlabelled DG to give a final concentration of 25 mM and 0·25 μCi/ml. Cell number was determined from replicate cultures and DG uptake was calculated per $10^6$ cells for 20 min.

*Induction of SCE.*—The cells were plated at a density of $10^4$ cells/ml (15 ml in T-30 flasks). After 48 h the culture medium was removed and an equal volume of medium containing 5-bromo-2′-deoxyuridine (BrdU, Sigma) and varying concentrations of test chemicals was added to the culture. Appropriate control cultures received either BrdU + 0·25% DMSO or BrdU alone. The cultures were kept in complete darkness to minimize the induction of exchanges caused by photolysis of BrdU containing DNA (Ikushima & Wolff, 1974) and were handled under a yellow safe light. After further incubation for 24 h, during which the cells went through 2 rounds of DNA replication, colcemid (0·02 μg/ml; GIBCO, Grand Island, N.Y.) was added to arrest the cells in mitosis, and 2 h later the cells were harvested using 0·05% trypsin. The cells were suspended in a 0·075M KCl hypotonic solution for 20 min, and then fixed in methanol:acetic acid (3:1) for 30 min. After 2 more changes of fixative, cells were spread on microscope slides and air-dried. A modified fluorochrome + Giemsa technique (Perry & Wolff, 1974) was used to stain the chromosomes. Metaphase plates of cells containing clearly differentiated chromosomes were examined under an oil immersion objective, using a Zeiss Universal Microscope. The SCEs in 50 cells selected at random were scored for each concentration of the test compounds in all the cell systems and experimental conditions used. Exchanges from 50 cells were also counted for every set of control experiments, to determine the baseline frequency of SCE, and the data were analysed statistically.

### RESULTS

From preliminary experiments, it was found that phenobarbitone and saccharin were not toxic to hamster cell lines up to doses of 100 μg/ml and 1 mg/ml respectively. On the other hand, TPA was toxic (reduced the colony-forming ability in the continuous presence of TPA for 10 days) even at 0·01 μg/ml, but the toxicity was not dose-dependent. In the presence of BrdU (a prerequisite for visualizing SCE) and where the duration of exposure to TPA was relatively short (24–30 h) no acute toxicity was seen, though there was a delay in progression through the cell cycle (Table I).

TABLE I.—*Effect of TPA on cell-cycle progression of V-79 cells maintained for 30 h in the presence of BrdU (10 μg/ml)*

| Medium | TPA (μg/ml) | % Mitosis I | II | III |
|---|---|---|---|---|
| F-10 | 0 | 4 | 56 | 40 |
| F-10 | 0·01 | 4 | 92 | 4 |
| F-10 | 1·0 | 6 | 82 | 12 |
| DMEM | 0 | 5 | 54 | 42 |
| DMEM | 0·01 | 4 | 87 | 11 |
| DMEM | 1·0 | 1 | 93 | 6 |

If the cells were cultured for 30 h, ~50% of the cells in the control culture entered into the 3rd mitosis (as evidenced by chromosomes heavily labelled with BrdU and with few segments containing the original thymidine). In the TPA-treated cultures, most of the cells remained in the 2nd mitosis. Since many cells in the 2nd mitosis were present after 24 h incubation of this asynchronized culture (both in the control and in the TPA-treated cells), it was not found necessary to keep the cells any longer in experiments where SCE was quantitated.

Treatment of CHO and the hybrid clone with TPA (lot 022) produced a significant increase of SCE over control at both concentrations tested (Table II). A second series of experiments was designed to investigate whether there is (a) any contribution by BrdU to TPA-induced SCE; (b) any dose-dependent increase in the TPA-induced SCE, or (c) whether the induction of SCE by TPA reflects minor impurities in the samples, as suggested by Loveday & Latt (1979). We looked at (a) two concentrations of BrdU (5 and 10 μg/ml), (b) a wider range of TPA doses and (c) a different batch of TPA (lot 026) which is currently being used by a number of research groups. The concentration of BrdU does not appear to modify the SCE of the control or TPA-treated cells (Table II; Expt 2). TPA gave a significant increase in the number of SCE (Table II) even at a dose as low as 0·01 μg/ml. A dose of 0·001 μg/ml produced no significant increase of SCE over the control value (data not shown). However, the SCE were not increased by higher doses (up to 1 μg/ml) and both batches of TPA gave similar results. We have checked not only the SCE induction by TPA in the main line of CHO, but also in a sub-clone. The TPA enhancement ratio of SCE in the clone was similar to that of the parent culture (Table II). We then tested the effect of TPA on the hamster lung cells, V-79, since the initial experiments of Kinsella & Radman (1978) were done with these cells. Moreover, the same cells were cultured either in F-10 or DMEM, since different culture media have been used by the different investigators. In both experiments TPA at 0·01 μg/ml produced a similar and significant increase in SCE; which was similar to that obtained with other indicator cells (Table II).

Experiments were then performed to study the effect of heat-in-activation of FCS on SCE induction. The production of SCE was enhanced in both the control, and in the TPA-treated cells when they were incubated with heat-inactivated FCS (Table III). However, the enhancement of SCE by TPA in the V-79 cells was the same, whether the FCS was heat-inactivated or not. When L-cysteine, a scavenger for free radicals, was added to the heat-inactivated serum, SCE in the control was unchanged. However, the enhancement ratio induced by TPA was lower in the presence of L-cysteine, showing that this

TABLE II.—*Effect of TPA sister chromatid exchanges in 4 cell lines under different experimental conditions*

| Expt | Cell line | Culture medium | Concentration of BrdV ($\mu$g/ml) | TPA* ($\mu$g/ml) | SCE/chromosome Mean ± s.e. | SCE/chromosome Range | Treated control† |
|---|---|---|---|---|---|---|---|
| 1 | CHO | Ham F-10 | 10 | 0 | 0·59±0·03 | 0·43–0·82 | |
| | | | | 0·1 | 0·86±0·04 | 0·53–1·21 | 1·44 |
| | | | | 1·0 | 0·87±0·05 | 0·55–1·29 | 1·46 |
| | Hybrid clone 3·1‡ | Ham F-10 | 10 | 0 | 0·36±0·02 | 0·22–0·60 | |
| | | | | 0·1 | 0·47±0·03 | 0·32–0·78 | 1·32 |
| | | | | 1·0 | 0·59±0·02 | 0·43–0·86 | 1·66 |
| 2 | CHO | Ham F-10 | 5 | 0 | 0·51±0·02 | 0·30–0·94 | |
| | | | | 0·01 | 0·64±0·02 | 0·36–1·11 | 1·25 |
| | | | | 0·1 | 0·67±0·02 | 0·35–1·26 | 1·30 |
| | | | | 0·3 | 0·68±0·02 | 0·42–1·15 | 1·33 |
| | | | | 1·000 | 0·67±0·03 | 0·38–1·15 | 1·31 |
| | | | 10 | 0 | 0·53±0·02 | 0·23–0·95 | |
| | | | | 0·01 | 0·69±0·02 | 0·40–1·25 | 1·30 |
| | | | | 0·1 | 0·73±0·03 | 0·30–1·66 | 1·37 |
| | | | | 0·3 | 0·71±0·02 | 0·31–1·50 | 1·34 |
| | | | | 1·0 | 0·67±0·02 | 0·42–1·11 | 1·26 |
| 3 | CHO Clone | Ham F-10 | 10 | 0 | 0·53±0·02 | 0·25–0·95 | |
| | | | | 0·3 | 0·76±0·03 | 0·40–2·17 | 1·44 |
| | | | | 1·0 | 0·82±0·02 | 0·45–1·31 | 1·56 |
| 4 | V-79 | Ham F-10 | 10 | 0 | 0·46±0·02 | 0·22–0·95 | |
| | | | | 0·01 | 0·67±0·03 | 0·35–1·20 | 1·46 |
| | | | | 1·0 | 0·60±0·02 | 0·42–1·09 | 1·30 |
| | | DMEM | 10 | 0 | 0·46±0·02 | 0·27–0·96 | |
| | | | | 0·01 | 0·60±0·03 | 0·29–1·29 | 1·32 |
| | | | | 1·000 | 0·68±0·02 | 0·33–1·19 | 1·49 |

* TPA in Expt 1 was from lot 022 and in Expts 2–4 from lot 026.
† Probability associated with tests of control *vs* treated using *t* test was always <0·0001.
‡ See Iype *et al.* (1981) for nomenclature of the hybrid clone of rat liver and hamster ovary cells.

TABLE III.—*SCE in V-79 cells under different serum conditions with and without TPA*

| FCS | Treatment | SCE/chromosome Mean ± s.e. | SCE/chromosome Range | Treated control | $P$* |
|---|---|---|---|---|---|
| Regular | None | 0·45±0·03 | 0·22–0·70 | | |
| | TPA (1 $\mu$g/ml) | 0·59±0·03 | 0·41–0·95 | 1·29 | <0·005 |
| Heat-inactivated | None | 0·59±0·03 | 0·35–1·08 | | |
| | TPA (1 $\mu$g/ml) | 0·79±0·03 | 0·49–1·16 | 1·34 | <0·001 |
| Heat-inactivated + L cysteine ($10^{-4}$ M) | None | 0·61±0·03 | 0·33–0·8 | | |
| | TPA (1 $\mu$g/ml) | 0·74±0·04 | 0·45–1·15 | 1·19 | <0·025 |

* Treated *vs* control in *t* test.

TABLE IV.—*Effect of TPA on carcinogen-induced SCE*

| Carcinogen | Concentration ($\mu$g/ml) | TPA (1 $\mu$g/ml) | SCE/chromosome Mean ± s.e. | SCE/chromosome Range | TPA treated untreated | $P$ ($t$ test) |
|---|---|---|---|---|---|---|
| — | — | − | 0·50±0·02 | 0·31–0·79 | | |
| | | + | 0·68±0·03 | 0·47–1·00 | 1·35 | <0·005 |
| MNNG | 0·05 | − | 1·90±0·12 | 1·20–3·25 | | |
| | | + | 1·94±0·14 | 1·19–3·47 | 1·02 | NS |
| 7-Bromomethyl-benz[*a*]anthracene | 0·1 $\mu$M | − | 1·08±0·05 | 0·61–1·58 | | |
| | | + | 1·21±0·06 | 0·60–1·68 | 1·12 | NS |

TABLE V.—*The effect of tumour promoters on the uptake of 2-deoxyglucose in V-79 cells*

| Promoter | Concentration (μg/ml) | DG uptake* pmol/$10^6$ cells/min (mean ± s.e.) | Treated/control |
|---|---|---|---|
| TPA | 0 | 119 ± 8 | |
| (lot 026) | 0·1 | 165 ± 4 | 1·40 |
| | 1·0 | 169 ± 8 | 1·43 |
| TPA | 0 | 136 ± 10 | |
| (+0·05 μg/ml | 0·1 | 170 ± 8 | 1·25 |
| MNNG) | 1·0 | 172 ± 4 | 1·26 |
| Saccharin | 0 | 94 ± 4 | |
| | 100 | 104 ± 4 | 1·11 NS |
| | 1000 | 134 ± 5 | 1·42 |
| Phenobarbital | 0 | 94 ± 4 | |
| | 10 | 91 ± 4 | 0·96 NS |
| | 100 | 112 ± 4 | 1·19 |

* The uptake of DG (0·25 μCi/ml; 25 nmol/ml) was studied for 20 min in PBS, as described by Siddiqi & Iype (1975).

free radical scavanger reduced TPA-enhanced SCE (Table III).

The effect of TPA on the higher levels of SCE pre-induced by carcinogens was then studied by treating V-79 cells first with low doses of MNNG or 7-bromomethylbenz[*a*]anthracene (30 min), and then with 1 μg/ml of TPA. Whilst TPA did enhance the SCE of the control cells, the SCE pre-induced by the carcinogens/mutagens was not further enhanced by TPA (Table IV).

Since TPA is known to produce a number of changes in cell-surface membranes, we have studied its effect on the uptake of DG in V-79 cells. It was found that TPA enhanced DG uptake ~1·4-fold, and the enhancement was similar with both concentrations (Table V). However, TPA still increased the DG uptake, even in cells pretreated with MNNG. Saccharin and phenobarbitone also showed an increase in DG uptake at the higher concentrations (Table V) but they also elicited a significant increase in SCE over the controls at lower concentrations (Table VI). As with TPA, no dose-dependent increase in SCE was seen when cells were treated with saccharin or phenobarbitone (Tables II, VI).

### DISCUSSION

Although considerable work has been reported on the effect of TPA on SCE induction, as mentioned earlier, the results were variable. We have studied a number of factors which might have produced these discrepancies.

An increase in SCE could result from prolonged treatment with BrdU (Ockey, 1980). TPA did produce a delay in the progression through cell cycle (Table I) and such temporary delay of growth has been observed in other cell systems (Peterson *et al.*, 1977, Kinzel *et al.*, 1981). We maintained the duration of BrdU-treatment constant at 24 h, though maintenance for longer could have produced even more SCE in the treated samples (Ockey, 1980). Having standardized the experimental conditions, the effect of different concentrations of BrdU and different batches of TPA was then studied (Table II) and we found no appreciable differences in the enhancement of SCE by TPA. However, there was no dose-dependency beyond the threshold concentration. This result is not consistent with

TABLE VI.—*Effect of saccharin or phenobarbitone on SCE in V-79 cells*

| Chemical | Concentration (μg/ml) | SCE/chromosme Mean ± s.e. | SCE/chromosme Range | Treated/control |
|---|---|---|---|---|
| — | — | 0·56 ± 0·02 | 0·28–0·95 | — |
| Saccharin | 100 | 0·74 ± 0·03 | 0·37–1·37 | 1·32 |
| | 1000 | 0·61 ± 0·03 | 0·33–1·09 | 1·10* |
| Phenobarbitone | 10 | 0·80 ± 0·03 | 0·45–1·34 | 1·43 |
| | 100 | 0·76 ± 0·02 | 0·54–1·05 | 1·36 |

* Statistically not significant in this sample, in all other samples the $P < 0·02$.

that of Nagasawa & Little (1981), who found a dose-dependent increase of SCE from 0·1 to 4 $\mu$g/ml with the same batch (lot 026) of TPA, but no increase with 0·01 $\mu$g/ml. The difference in our results may be due to the different indicator cells used.

It is possible that different cell lines have inherent differences of sensitivity to SCE production. Recently, a CHO cell line with a high base-line frequency of SCE was reported (Thompson *et al.*, unpub.). Using a sub-clone of CHO, as well as V-79 cells, we found a similar enhancement of SCE to that seen in other cell types (Table II).

Nagasawa & Little (1981) reported that the induction of SCE by TPA was markedly suppressed in synchronized CHO cells if the FCS in the incubation medium was not heat-inactivated. We found that heat inactivation did not affect the enhancement of SCE (Table III).

Nagasawa & Little (1981) also reported that TPA-induced SCE with heat-inactivated serum could be reduced if superoxide dismutase was added to the medium. They and others (Emerit & Cerutti, 1981) have suggested that free radicals may be important intermediates in the induction of SCE by TPA. Recently Slaga *et al.* (1981) also reported that the generation of free radicals could lead directly or indirectly to membrane peroxidation. Our results with L-cysteine (Table III) suggest that it is likely that the action of TPA may be manifested through free-radical formation. However, even in the presence of L-cysteine, at a concentration which protects cells from chromosome damage (Emerit *et al.*, 1974; Raj & Heddle, 1980), there was still a significant enhancement of SCE by TPA (Table III). Therefore some other cellular properties altered by TPA may be at least partly responsible for its enhancement of SCE.

It has been shown that MNNG-induced SCE (Popescu *et al.*, 1980) or MNNG-induced forward mutagenesis (Kinsella, 1981) were not further affected by TPA in hamster embryo cells and V-79 cells, respectively. Gentil *et al.* (1980) also found that TPA does not increase SCE, when V-79 cells treated first with MNNG, were treated again with TPA, though they did observe SCE induction when TPA preceded MNNG treatment. From our experiments (Table IV) it is clear that the two direct-acting carcinogens/mutagens considerably increased the level of SCE 2–4-fold, and that it was not further enhanced by TPA.

TPA is known to produce a number of changes in the cell-surface membranes (Wenner *et al.*, 1974; Blumberg *et al.*, 1976; Wigler & Weinstein, 1976; Dridger & Blumberg, 1977; Lee & Weinstein, 1978; Shoyab *et al.*, 1979; Fisher *et al.*, 1979). Dridger & Blumberg (1977) reported a marked increase in DG uptake in "resting" chick embryo fibroblasts after TPA. However, even in cells grown in 10% serum, the DG uptake (which was 7·4-fold higher than that of resting cells) was further increased 1·4-fold by TPA. Our experiments with V-79 cells were comparable with the latter condition used by Dridger & Blumberg (1977) and we observed a similar enhancement (Table V). Since the enhancement ratios of DG uptake and SCE production (Table II) were similar, it is possible that the increase in SCE by TPA may be effected through alterations in the properties of cell-surface membranes. However, under conditions where there was no further increase in SCE by TPA (*i.e.* cells pretreated with MNNG), this promoter still increased DG uptake (Table V). This may be because MNNG *per se* did not significantly affect cell permeability. On the other hand, if the increase in cell-membrane permeability due to TPA is partly responsible for enhancing SCE in the control cells, it does not appear to do so in the carcinogen-treated V-79 cells. As the increased SCE produced by mutagens (primarily due to inter-action with DNA) is great, the small enhancement of SCE by TPA through the mechanisms suggested (*via* free-radical formation and/or alteration in cell-membrane permeability) may not be manifested. This is consistent with the finding of DiPaolo *et al.* (1980) that SCE preinduced by MNNG was also not affected by antipain, an inhibitor of cell-surface protease.

Saccharin has been shown to induce SCE in CHO cells by Wolff & Rodin (1978) without any dose-dependent change between 1 and 10 mg/ml. We used 0·1 and 1 mg/ml on V-79 cells and found the same enhancement as reported by these authors for CHO cells. A much higher dose of saccharin than TPA was needed to induce SCE. Trosko *et al.* (1980) have shown that 0·1 $\mu$g/ml TPA inhibited metabolic cooperation between cells more than did 5 mg/ml of saccharin. However, both agents change the properties of the cell membrane. It can also be seen (Table V) that saccharin at 1 mg/ml increased the DG uptake to a level similar to that produced by low doses of TPA.

There have been no previous studies on SCE induction by the liver-tumour promoter phenobarbitone. We found that it significantly enhanced SCE (Table VI) even at a concentration of 10 $\mu$g/ml. The membrane permeability was not affected at this level (Table V). At 100 $\mu$g/ml the uptake of DG was also significantly increased. Phenobarbitone is known to alter membrane-associated enzymes, which may be related to its promoting activity (Williams *et al.*, 1980). As with TPA and saccharin, SCE enhancement by phenobarbitone was not altered by the 10-fold increase in dose (Tables II, VI).

We have shown earlier that, in our cell systems, SCE are induced by mutagenic carcinogens (Ray-Chaudhuri *et al.*, 1980; Iype, *et al.*, 1981) that are known to interact with DNA. Methapyrilene and nitrosodiethanolamine, which are non-mutagenic carcinogens, were incapable of producing SCE (Iype *et al.*, unpublished). Despite their varied and numerous biological properties, tumour promoters (especially TPA) are not known to interact directly with DNA; yet TPA, saccharin and phenobarbitone did enhance SCE formation; thus they must act by some mechanism other than direct DNA interaction. These agents may indirectly perturb DNA *via* free-radical formation, as suggested earlier (Nagasawa & Little, 1981; Slaga *et al.*, 1981) which may be partly responsible for the SCE enhancement by TPA shown in this study. An important difference between SCE induction by mutagenic carcinogen and tumour promoters is that in the former the induction increases with the dose of the agent, whereas in the latter there is an "all or none" effect after a threshold dose. Such an effect may be mediated through the change in the cell membrane produced at the threshold dose, and above, of the promoters.

Another factor involved in the enhancement of SCE by TPA may be the delay in the progression of the cell cycle. Since chromatid exchanges are formed during S phase of the cell cycle, it is likely that any agent which lengthened this phase would also increase the *basal level* of SCE seen in cultured cells.

From these studies, it is clear that TPA did enhance SCE production in 3 indicator-cell systems under different experimental conditions. The lack of SCE enhancement reported by some other investigators is therefore unlikely to be due to variables such as batch of TPA, culture medium and heat inactivation of the serum. However, different cell lines may not always respond similarly to TPA. In the cells used in our experiments, saccharin and phenobarbitone also enhanced SCE.

The authors wish to thank Susan Kelley and Albert Herring for their contributions. This work was supported by Contract N01-CO-75380 with the National Cancer Institute.

### REFERENCES

Blumberg, P. M., Driedger, P. E. & Rossow, P. W. (1976) Effect of a phorbol ester on a transformation-sensitive surface protein of chick fibroblasts. *Nature*, **264**, 446.

Bradley, M. O., Hsu, I. C. & Harris, C. C. (1979) Relationships between sister chromatid exchange and mutagenicity, toxicity and DNA damage. *Nature*, **282**, 318.

Cassell, D. M. & Latt, S. A. (1980) Relationship between DNA adduct formation and sister chromatid exchange induction by [$^3$H]8-Methoxypsoralen in Chinese hamster ovary cells. *Exp. Cell Res.*, **128**, 15.

CONNELL, J. R. & DUNCAN, S. J. (1981) The effect of non-phorbol promoters as compared with phorbol myristate acetate on sister chromatid exchange induction in cultured Chinese hamster cells. *Cancer Letters*, **11**, 351.

DIAMOND, L., O'BRIEN, T. G. & ROVERA, G. (1978) Tumor promoters: Effects on proliferation and differentiation of cells in culture. *Life Sci.*, **23**, 1979.

DIPAOLO, J. A., AMSBAUGH, S. C. & POPESCU, N. C. (1980) Antipain inhibits N-methyl-N′-nitro-N-nitrosoguanidine-induced transformation and increases chromosomal aberrations. *Proc. Natl Acad. Sci.*, **77**, 6649.

DRIDGER, P. E. & BLUMBERG, P. M. (1977) The effect of phorbol diesters on chicken embryo fibroblasts. *Cancer Res.*, **37**, 3257.

EMERIT, I. & CERUTTI, P. A. (1981) Tumor promoter phorbol-12-myristate-13-acetate induces chromosomal damage *via* indirect action. *Nature*, **293**, 144.

EMERIT, I., LEVY, A. & HOUSSET, E. (1974) Breakage factor in systemic sclerosis and protector effect of L-cysteine. *Humangenetik*, **25**, 221.

FISHER, P. B., FLAMM, M., SCHACHTER, D. & WEINSTEIN, I. B. (1979) Tumor promoters induce membrane changes detected by fluorescence polarization. *Biochem. Biophys. Res. Comm.*, **86**, 1063.

FUKUSHIMA, S., FRIEDELL, G. H., JACOBS, J. B. & COHEN, S. M. (1981) Effect of L-tryptophan and sodium saccharin on urinary tract carcinogenesis initiated by N-[4-(5-nitro-2-furyl)-2′thiazolyl]-formamide. *Cancer Res.*, **41**, 3100.

GENTIL, A., RENAULT, G. & MARGOT, A. (1980) The effect of the tumor promoter 12-O-tetradecanoyl-phorbol-13-acetate (TPA) on UV and MNNG-induced sister chromatid exchanges in mammalian cells. *Int. J. Cancer*, **26**, 517.

HAM, R. G. (1963) An improved nutrient solution for diploid Chinese hamster and human cell lines. *Exp. Cell Res.*, **29**, 515.

HSUEH, J. L., CHEN, H. H. & HUANG, C. C. (1981) Effect of tumor promoter 12-O-tetradecanoyl-phorbol-13-acetate on induction of sister-chromatid exchanges in Chinese hamster V79 cells treated with mutagens. *Mutat. Res.*, **81**, 387.

IKUSHIMA, T. & WOLFF, S. (1974) Sister chromatid exchanges induced by light flashes to 5-bromodeoxyuridine and 5-iododeoxyuridine substituted Chinese hamster chromosomes. *Exp. Cell Res.*, **87**, 15.

IYPE, P. T., RAY-CHAUDHURI, R. & KELLEY, S. (1981) Hybrid clones of rat liver and hamster ovary cells as targets for carcinogen screening. *Carcinogenesis*, **2**, 49.

KANO, Y. & FUJIWARA, Y. (1981) Roles of DNA interstrand crosslinking and its repair in the induction of sister-chromatid exchange and a higher induction in Fanconi's anemia cells. *Mutat. Res.*, **81**, 365.

KINSELLA, A. R. (1981) Investigation of the effects of the phorbol ester TPA on carcinogen-induced forward mutagenesis to 6-thioguanine-resistance in V-79 Chinese hamster cells. *Carcinogenesis*, **2**, 43.

KINSELLA, A. R. & RADMAN, M. (1978) Tumor promoter induces sister chromatid exchanges: Relevance to mechanisms of carcinogenesis. *Proc. Natl Acad. Sci.*, **75**, 6149.

KINZEL, V., RICHARDS, J. & STÖHR, M. (1981) Responses of synchronized HeLa cells to the tumor-promoting phorbol ester 12-O-tetradecanoylphorbol-13-acetate as evaluated by flow cytrometry. *Cancer Res.*, **41**, 306.

LEE, L. S. & WEINSTEIN, I. B. (1978) Tumor promoting phorbol esters inhibit binding of epidermal growth factor to cellular receptors. *Science*, **202**, 313.

LITTLE, J. B., NAGASAWA, H. & KENNEDY, A. R. (1979) DNA repair and malignant transformation: Effect of X-irradiation, 12-O-tetradecanoylphorbol-13-acetate, and protease inhibitors on transformation and sister chromatid exchange in mouse 10T-1/2 cells. *Radiat. Res.*, **79**, 241.

LOVEDAY, K. S. & LATT, S. A. (1979) The effect of a tumor promoter, 12-O-tetradecanoyl-phorbol-13-acetate (TPA) on sister chromatid exchange formation in cultured Chinese hamster cells. *Mutat. Res.*, **67**, 343.

MILLER, R. C., GEARD, C. R., OSMAK, R. S. & 7 others (1981) Modification of sister chromatid exchanges and radiation-induced transformation in rodent cells by the tumor promoter 12-O-tetradecanoylphorbol-13-acetate and two retinoids. *Cancer Res.*, **41**, 655.

MONDAL, S., BRANKOW, D. W. & HEIDELBERGER, C. (1978) Enhancement of oncogenesis in C3H/10T1/2 mouse embryo cell cultures by saccharin. *Science*, **201**, 1141.

MORTON, H. J. (1970) Survey of commercially available tissue culture media. *In Vitro*, **6**, 89.

NAGASAWA, H. & LITTLE, J. B. (1979) Effect of tumor promoters, protease inhibitors, and repair processes on X-ray-induced sister chromatid exchanges in mouse cells. *Proc. Natl Acad. Sci.* **76**, 1943.

NAGASAWA, H. & LITTLE, J. B. (1981) Factors influencing the induction of sister chromatid exchanges in mammalian cells by 12-O-tetradecanoylphorbol-13-acetate. *Carcinogenesis*, **2**, 601.

NATARAJAN, A. T., TATES, A. D., VAN BUUL, P. P. W., MEIJERS, M., & DE VOGEL, N. (1976) Cytogenetic effects of mutagens/carcinogens after activation in microsomal system *in vitro*. I. Induction of chromosome aberrations and sister chromatid exchanges by diethylnitrosamine (DEN) and dimethylnitrosamine (DMN) in CHO cells in the presence of rat liver microsomes. *Mutat. Res.*, **37**, 83.

OCKEY, C. H. (1980) Differences between "spontaneous" and induced sisterchromatid exchanges with fixation time and their chromosome localization. *Cytogenet. Cell Genet.*, **26**, 223.

PERAINO, C., FRY, R. J. M. & STAFFELDT, E. (1977) Effects of varying the onset and duration of exposure to phenobarbital on its enhancement of acetylaminofluorene-induced hepatic tumorigenesis. *Cancer Res.*, **37**, 3623.

PERRY, P. & EVANS, H. J. (1975) Cytological detection of mutagen-carcinogen exposure by sister chromatid exchange. *Nature*, **258**, 121.

PERRY, P. & WOLFF, S. (1974) New Giemsa method for the differential staining of sister chromatids. *Nature*, **251**, 156.

PETERSON, A. R., MONDAL, S., BRANKOW, D. W., THON, W. & HEIDELBERGER, C. (1977) Effects of promoters on DNA synthesis in C3H/10T1/2 mouse fibroblasts. *Cancer Res.*, **37**, 3223.

POPESCU, N. C., AMSBAUGH, S. C. & DIPAOLO, J. A. (1980) Enhancement of N-methyl-N′-nitro-N-nitrosoguanidine transformation of Syrian hamster cells by a phorbol diester is independent of sister chromatid exchanges and chromosome aberrations. *Proc. Natl Acad. Sci.*, **77**, 7282.

RAY-CHAUDHURI, R., KELLEY, S. & IYPE, P. T. (1980) Induction of sister chromatid exchanges by carcinogens mediated through cultured rat liver epithelial cells. *Carcinogenesis*, **1**, 779.

RAJ, A. S. & HEDDLE, J. A. (1980) The effect of superoxide dismutase, catalase and L-cysteine on spontaneous and on mitomycin C induced chromosomal breakage in Fanconi's anemia and normal fibroblasts as measured by the micronucleus method. *Mutat. Res.*, **78**, 59.

SHOYAB, M., DE LARCO, J. E. & TODARO, G. J. (1979) Biologically active phorbol esters specifically alter affinity of epidermal growth factor membrane receptors. *Nature*, **279**, 387.

SIDDIQI, M. & IYPE, P. T. (1975) Studies on the uptake of 2-deoxy-D-glucose in normal and malignant rat epithelial liver cells in culture. *Int. J. Cancer*, **15**, 773.

SLAGA, T. J., KLEIN-SZANTO, A. J. P., TRIPLETT, L. L. & YOTTI, L. P. (1981) Skin tumor-promoting activity of benzoyl peroxide, a widely used free radical-generating compound. *Science*, **213**, 1023.

SWENSON, D. H., HARBACH, P. R. & TRZOS, R. J. (1980) The relationship between alkylation of specific DNA bases and induction of sister chromatid exchange. *Carcinogenesis*, **1**, 931.

THOMPSON, L. H., BAKER, R. M., CARRANO, A. V. & BROOKMAN, K. W. (1980) Failure of the phorbol ester 12-O-tetradecanoylphorbol-13-acetate to enhance sister chromatid exchange, mitotic segregation, or expression of mutations in Chinese hamster cells. *Cancer Res.*, **40**, 3245.

TROSKO, J. E., CHANG, C., YOTTI, L. P. & CHU, E. H. Y. (1977) Effect of phorbol myristate acetate on the recovery of spontaneous and ultraviolet light induced 6-thioguanine and ouabain-resistant Chinese hamster cells. *Cancer Res.*, **37**, 188.

TROSKO, J. E., DAWSON, B., YOTTI, I. P. & CHANG, C. C. (1980) Saccharin may act as a tumor promoter by inhibiting metabolic cooperation between cells. *Nature*, **285**, 109.

WENNER, C. E., HACKNEY, J., KIMELBERG, H. K. & MAYHEW, E. (1974) Membrane effects of phorbol esters. *Cancer Res.*, **34**, 1731.

WIGLER, M. & WEINSTEIN, I. B. (1976) Tumor promotor induces plasminogen activator. *Nature*, **259**, 232.

WILLIAMS, G. M., OHMORI, T., KATAYAMA, S. & RICE, J. M. (1980) Alteration by phenobarbital of membrane-associated enzymes including gamma glutamyl transpeptidase in mouse liver neoplasms. *Carcinogenesis*, **1**, 813.

WOLFF, S. & RODIN, B. (1978) Saccharin-induced sister chromatid exchanges in Chinese hamster and human cells. *Science*, **200**, 543.